# Impact of *Dinophysis acuminata* Feeding *Mesodinium rubrum* on Nutrient Dynamics and Bacterial Composition in a Microcosm

**DOI:** 10.3390/toxins10110443

**Published:** 2018-10-30

**Authors:** Han Gao, Chenfeng Hua, Mengmeng Tong

**Affiliations:** Ocean College, Zhejiang University, No 1 Zheda Road, Zhoushan 316000, Zhejiang, China; gghanbing@zju.edu.cn (H.G.); verahcf@zju.edu.cn (C.H.)

**Keywords:** DSP toxins, pectenotoxins, *Dinophysis acuminata*, *Mesodinium rubrum*, bacterial community, high throughput sequencing

## Abstract

The development of *Dinophysis* populations, producers of diarrhetic shellfish toxins, has been attributed to both abiotic (e.g., water column stratification) and biotic (prey availability) factors. An important process to consider is mixotrophy of the *Dinophysis* species, which is an intensive feeding of the *Mesodinium* species for nutrients and a benefit from kleptochloroplasts. During the feeding process, the nutritional status in the environment changes due to the preference of *Mesodinium* and/or *Dinophysis* for different nutrients, prey cell debris generated by sloppy feeding, and their degradation by micro-organisms changes. However, there is little knowledge about the role of the bacterial community during the co-occurrence of *Mesodinium* and *Dinophysis* and how they directly or indirectly interact with the mixotrophs. In this study, laboratory experiments were performed to characterize the environmental changes including those of the prey present, the bacterial communities, and the ambient dissolved nutrients during the co-occurrence of *Mesodinium rubrum* and *Dinophysis acuminata*. The results showed that, during the incubation of the ciliate prey *Mesodinium* with its predator *Dinophysis*, available dissolved nitrogen significantly shifted from nitrate to ammonium especially when the population of *M. rubrum* decayed. Growth phases of *Dinophysis* and *Mesodinium* greatly affected the structure and composition of the bacterial community. These changes could be mainly explained by both the changes of the nutrient status and the activity of *Dinophysis* cells. *Dinophysis* feeding activity also accelerated the decline of *M. rubrum* and contamination of cultures with okadaic acid, dinophysistoxin-1, and pectenotoxin-2, but their influence on the prokaryotic communities was limited to the rare taxa (<0.1%) fraction. This suggests that the interaction between *D. acuminata* and bacteria is species-specific and takes place intracellularly or in the phycosphere. Moreover, a majority of the dominant bacterial taxa in our cultures may also exhibit a metabolic flexibility and, thus, be unaffected taxonomically by changes within the *Mesodinium-Dinophysis* culture system.

## 1. Introduction

The cosmopolitan dinoflagellate species *Dinophysis acuminata* is responsible for diarrhetic shellfish poisoning (DSP) events all around the world [1,2]. Okadaic acid (OA) and its derivatives known as dinophysistoxins (DTXs) and/or pectenotoxins (PTXs) are the dominant components in the toxin profile of *D. acuminata*. As strong inhibitors of serine and threonine protein phosphatases in eukaryotic organisms, OA and DTXs are capable of promoting potent tumors [3], inducing typical diarrhetic symptoms [2,4], and even acting as lethal agents to mammals. Recent transcriptomics analysis also revealed that OA and DTX-1 may induce hypoxia-related pathways or processes, unfolded protein response (UPR), and endoplasmic reticulum (ER) stress [5]. PTXs are generally not responsible for unpleasant gastrointestinal symptoms but are potentially involved in acute toxicity [6].

*D. acuminata* is a mixotrophic species that primarily requires phototrophic metabolism and plastid retention for long-term maintenance in the laboratory [7,8,9]. The *Dinophysis*–*Mesodinium*–cryptophyte is so far the only known food chain for *Dinophysis* growth. *Dinophysis* blooms are very much related to the distribution and abundance of *Mesodinium* [10,11,12]. Therefore, the nutritional status of prey and the surrounding environment may have a critical impact on the growth and toxin production of *Dinophysis* [13,14,15,16,17]. The feeding process of the latter involves not only the direct uptake of the prey organelles through a feeding peduncle (myzocytosis) and secretion of mucus traps but also the intense lysis of the ciliate cells [18,19,20]. Cell debris and organic substances originating from prey were reported to induce the DSP toxin release from *Dinophysis* [21]. The suspected harmful compounds (e.g., free polyunsaturated fatty acids) were not the shellfish toxins [22]. Additionally, “sloppy feeding” behavior generates a substantial amount of dissolved and particulate materials in the surrounding environment. This pool of biological organic matter combined with the extracellular toxin fraction may also function as a source of nutrients available to the heterotrophic bacterial community and, in turn, for *Dinophysis* cells after regeneration [21,23,24] or other biochemical pathways [25]. However, few studies have been conducted to assess the contribution and availability of these nutritional sources.

The role of algal–bacterial interactions during harmful algal bloom (HAB) has received attention in recent years [26,27,28]. The supply of dissolved organic substances through cell exudation or cell lysis is hypothesized to be a major interaction between phytoplankton and the associated bacterial community [24,25,29]. The influence of bacteria on the toxigenic properties of photosynthetic microalgae (mainly *Alexandrium* spp. producing paralytic shellfish toxins) has been widely examined (Reference [26] and literature therein). The “obligate” relationship between bacteria and mixotrophic *Dinophysis* species has been explored in terms of cell abundance and carbon equivalents, which show a possible dependence on bacteria-produced vitamin B_12_ and, to a lesser extent, the potential of bacterivory for *Dinophysis* growth [23], which was otherwise confirmed in the case of *Mesodinium rubrum* [30]. Recently [31], the cluster of Alteromonadales have been identified as the unique prokaryotic microbiome associated with *D. acuminata* blooms in Northport Harbor, New York. This finding highlighted the importance of biogeochemical conditions in shaping the microbial consortia.

Mixotrophs may become the major players in an aquatic ecosystem due to their substantial contribution to the energy cycles and to nutrient cycles where heterotrophic bacteria control most of the pathways [32,33]. However, more compelling evidence is needed to explain the interactions between specific heterotrophic bacteria and nutrient dynamics mediated by the mixotrophy of *Dinophysis* species. Therefore, in this study, we focused on the bacterial community associated with the mixotrophic *D. acuminata* feeding on the mixotrophic *M. rubrum* in laboratory culture conditions. By tracking the changes of the bacterial assemblages and the nutritional status of the culture medium, we aimed to (i) study the nutrient dynamics mediated by mixotrophy through the different growth phases of *Dinophysis* feeding *Mesodinium* and the possible consequences for the ambient microbial community and (ii) identify the prevailing interactions among deterministic factors, the bacterial community, and DSP toxin dynamics during the feeding process. In the context of its mixotrophic nature, we hypothesized that an ingestion-derived nutrient shift combined with the activities of the toxin-producing *Dinophysis* could lead to niche separation of the microbial community.

## 2. Results

### 2.1. Predator-Prey Population Dynamics and Environmental Changes

**Figure 1:**
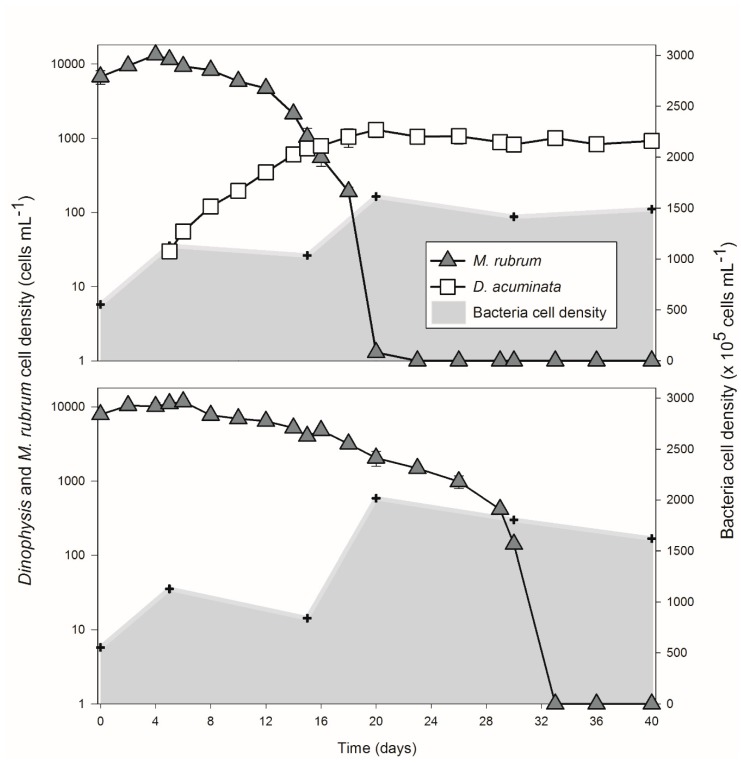
Cell density of *Mesodinium rubrum* and *Dinophysis acuminata* in Group A and Group B (control group without *Dinophysis* cells) over the growth curve. The shaded area indicates the bacterial concentration and the hex symbol shows the time point when subsamples were retrieved for nutrients analysis, bacterial counts, and analysis of the bacterial community.

The simulation started with an initial density of *M. rubrum* of 6740 ± 1379.3 (mean ± SD, Group A) cells mL^−1^ and 7902 ± 373.0 (Group B) cells mL^−1^ (Figure 1). The *M. rubrum* population developed until the 5th day when *Dinophysis* cells were inoculated (Group A). The ciliate cell-density gradually declined under an average ingestion rate of 3.25 ± 0.38 prey cells predator^−1^ day^−1^ during 16 days while the *Mesodinium* population in the control group (Group B) doubled in 4 days and then significantly declined to 4833 ± 378.6 cell mL^−1^ from days 5 to 16. *Dinophysis* exponential growth lasted 15 days (from T1 to T3) and reached a maximal density of 1302 ± 282.1 cells mL^−1^ on the 20th day and remained in a plateau phase thereafter (Figure 1). The bacterial abundance also changed during the feeding process (Group A) and the growth of *M. rubrum* (Group B).

Environmental characteristics varied differently over the course of the growth curve (Table 1). Based on our design, inorganic nitrogen (mainly comprised of NO_3_^−^) and phosphate PO_4_^3−^ concentrations at T0 (45.95 ± 5.77 and 2.90 ± 0.85 μM, respectively) were much lower than those in f/20 medium. As the population developed, a sharp increase of NH_4_^+^ was observed from T1 to T2 while NO_3_^−^ became undetectable (Table 1). PO_4_^3−^ also decreased but at a relatively slower rate. Accordingly, particulate phosphorus exhibited an opposite pattern and slightly declined after 30 days of incubation. Dissolved organic carbon (DOC) increased along with the rise and cell maxima of *M. rubrum.* Thereafter, DOC content was generally reduced with fluctuations. Particulate organic carbon (POC) remained constant for the first 5 days and decreased as the population declined (Table 1 and Figure 1). A separate trend of POC, however, was observed for T3 when this compound continued declining in Group B but gradually accumulated in Group A. In addition, toxins accumulated in the culture medium as the *Dinophysis* population increased at the expense of *Mesodinium* in Group A. OA and DTX1 were presented together in the summary and in the following analysis due to the fact that they share the same chemical backbone (Table 1). Total OA + DTX1 and PTX2 contents in the culture medium were shown to increase over the growth curve and reached a concentration of 4109.58 ± 621.79 pg mL^−1^ and 26.74 ± 0.73 ng mL^−1^, respectively, by the end of the experiment (Table 1).

### 2.2. Composition and Structure of the Microbial Community throughout the Growth Curve

**Figure 2:**
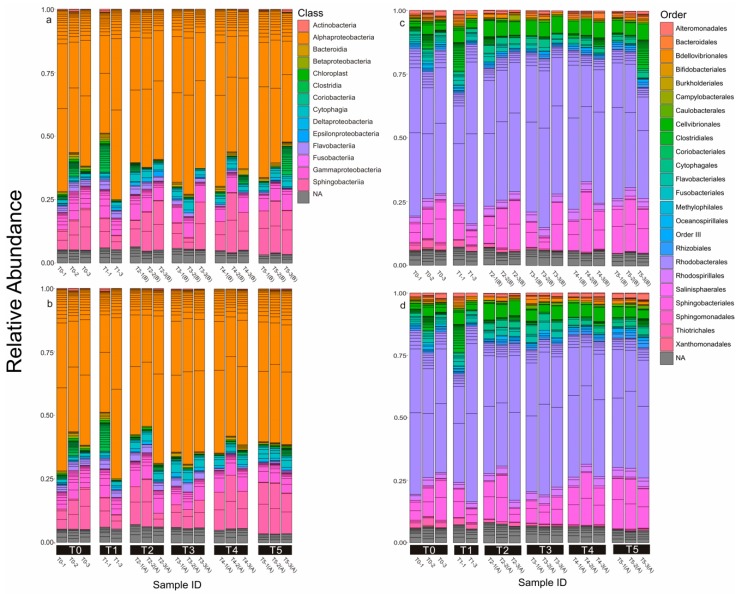
Microbial community compositions across all the samples are shown. Above the sample IDs, the time series of those samples were identified (T0–T5). The color key represents bacterial taxa at class (**a**,**b**) and order level (**c**,**d**).

**Figure 3:**
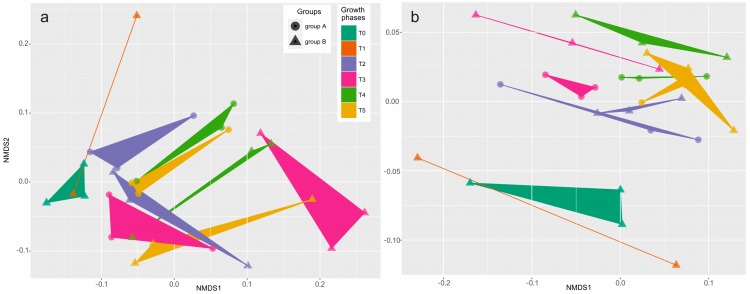
NMDS plot based on unweighted (**a**) and weighted (**b**) UniFrac distances. Sample points are shaded by growth phases and their shape represents groups. Growth phases seem to be more powerful in shaping the composition (ANOSIM, r = 0.257, *p* = 0.004) and structure (ANOSIM, r = 0.37, *p* = 0.001) of the communities compared to the presence/absence of *Dinophysis* cells (ANOSIM, unweighted r = −0.015, *p* = 0.544 and weighted r = −0.06, *p* = 0.933).

**Figure 4:**
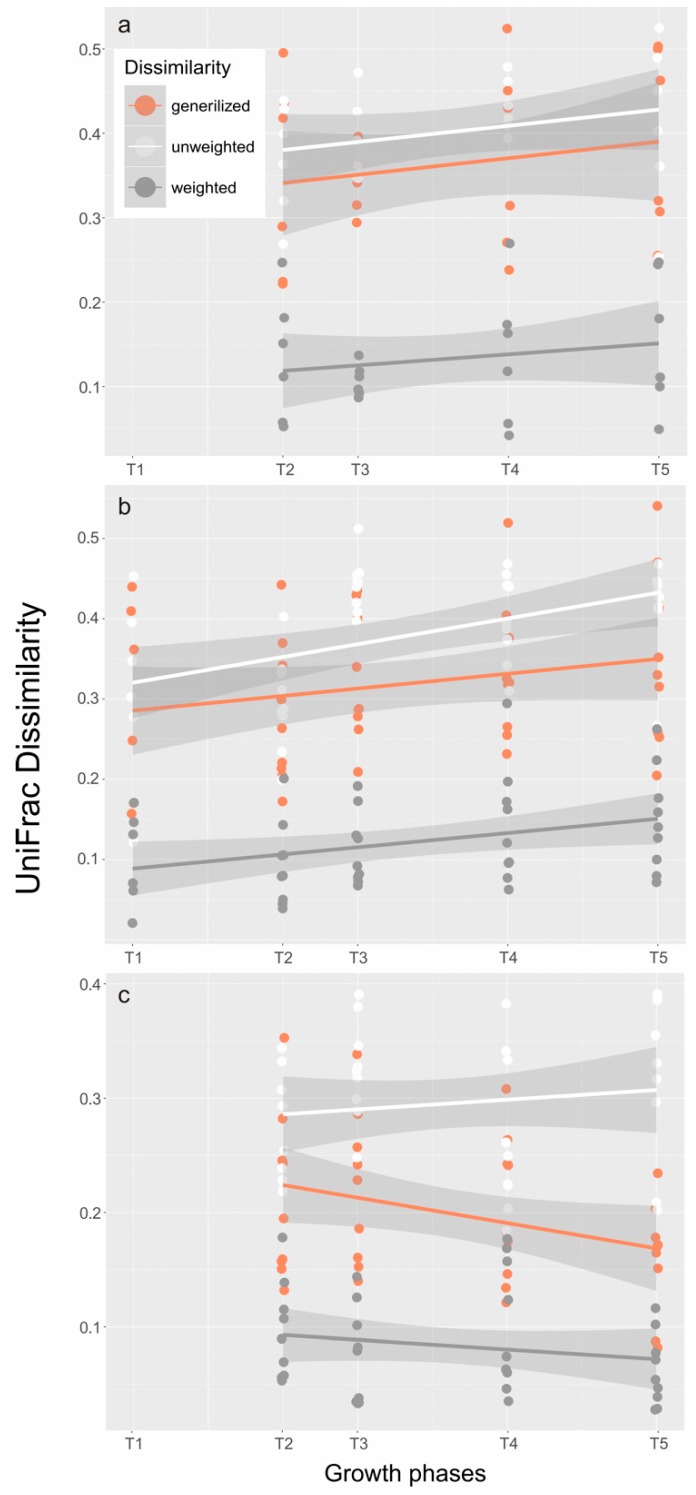
UniFrac dissimilarities between the onset and each of the following growth phases in (**a**) *Dinophysis* group (since *Dinophysis* cells were added at T1, calculations were carried out from T2) and (**b**) *M. rubrum* control group, and (**c**) dissimilarities between the two groups at the same phase were also plotted against growth phases. Three measures (unweighted, weighted, and generalized) were color-coded and liner fitted with a 95% confidence interval.

Bacteria samples were also harvested six times throughout the entire growth curve. The composition and structure of the microbial community was demonstrated at the class and order levels, according to the DNA results (Figure 2), and analyzed statistically using unweighted (structure) and weighted (composition) NMDS (Figure 3) and UniFrac dissimilarity (Figure 4).

During the molecular analysis, a total of 93 OTUs were observed throughout all the samples after being rarefied to an even depth of 25,396 reads (Table 2). A Good’s coverage index of over 0.999 and the high validity of clean tags indicated that the sequencing had covered almost all the species in the samples and the results were convincing. Alpha diversity indexes (Simpson, Shannon Wiener, and Chao1) indicated that there were no significant differences (ANOVA, *p* > 0.05) of the bacterial community between the two treatments in each crucial time period (Table 2). Proteobacteria (relative abundance = 74.3%) and Bacteroidetes (relative abundance = 21.1%) were the two dominant bacterial phyla in all samples. The majority of Proteobacteria were Alphaproteobacteria (97.8%) and a small fraction of Gammaproteobacteria (2.1%). Bacteroidetes and Sphingobacteria only attributed to the Sphingobacteriales accounted for 14.2% of the total microbial assemblage (Figure 2a). At the order level, Rhodobacterales and Cellvibrionales, which are representative of Alphaproteobacteria and Gammaproteobacteria, respectively, were dominant (Figure 2b).

NMDS also demonstrated the difference of structure (Figure 3a) and composition (Figure 3b) of the bacterial community in Groups A and B. The unweighted (Figure 3a) and weighted (Figure 3b) UniFrac distances represent the structure and composition of the microbial assemblage, respectively. The patterns of structural differences (Figure 3a) in both treatments were not as clear as the patterns of composition differences (Figure 3b) within the growth phases (T0–T5). In fact, growth phases (*Dinophysis* in Group A and *Mesodinium* in Group B) had a great effect on shaping the bacterial structure (ANOSIM r = 0.37, *p* = 0.001) and composition (ANOSIM r = 0.257, *p* = 0.004) of the communities regardless of whether *Dinophysis* cells were present or not (ANOSIM, unweighted r = −0.015, *p* = 0.544 and weighted r = −0.06, *p* = 0.933). The same results were also found in UniFrac dissimilarities analysis, which showed that the composition and structure of the bacterial community significantly changed during *Dinophysis* growth (Figure 4a) and *M. rubrum* decay (Figure 4b) by using the dissimilarity indexes and the generalized, unweighted, and weighted UniFrac distances. As for the difference between the two treatments (Figure 4c), only a minor increase in an unweighted distance analysis was observed and an opposite trend was observed when the abundance of bacterial taxa was considered (generalized and weighted UniFrac distance), which suggests that the presence and proliferation of the *Dinophysis* population or the consequences of its association with *Mesodinium* decay may not influence the composition and structure of the dominant bacterial species.

To better identify the interactions between the microbiome and the biotic or abiotic factors characterized in the growth curve, we manually defined the bacteria into three groups, which include the abundant (Ab) group, the moderately abundant (M) group, and the rare (R) taxa group. These groups stand for a relative abundance above 1%, between 0.1% and 1%, and below 0.1%, respectively. In summary, out of the 93 identified OTUs, 5 OTUs were assigned to the abundant (Ab) group, 83 OTUs met the criterion of rare taxa (R), and 5 OTUs belonged to the moderate (M) group.

The response of the microbial communities to the environmental changes was further investigated by the Mantel test (Table 3) and the most related factors were selected by the BIOENV procedure (Table 4). The results showed that all three assemblages were significantly correlated (*p* < 0.01) to the environmental matrix (Table 1) especially the M and R (r > 0.5), which suggests that the selected parameters had a better interpretation on a relatively lower abundance of bacterial assemblages. Cell density of *M. rubrum* and *D. acuminata* were also included in the “environmental matrix” (Table 1) for interpretation through the BIOENV procedure (Table 4). Out of the 11 parameters, eight were finally selected by BIOENV for the best correlation models to interpret the Bray–Curtis distance matrices of bacterial communities. They were *M. rubrum* density, *Dinophysis* cell density, DOC, PO_4_^3−^, NH_4_^+^, POP, OA + DTX1, and PTX2. Similarly, the selected parameters were more powerful in representing the M and R taxa (Table 4).

**Figure 5:**
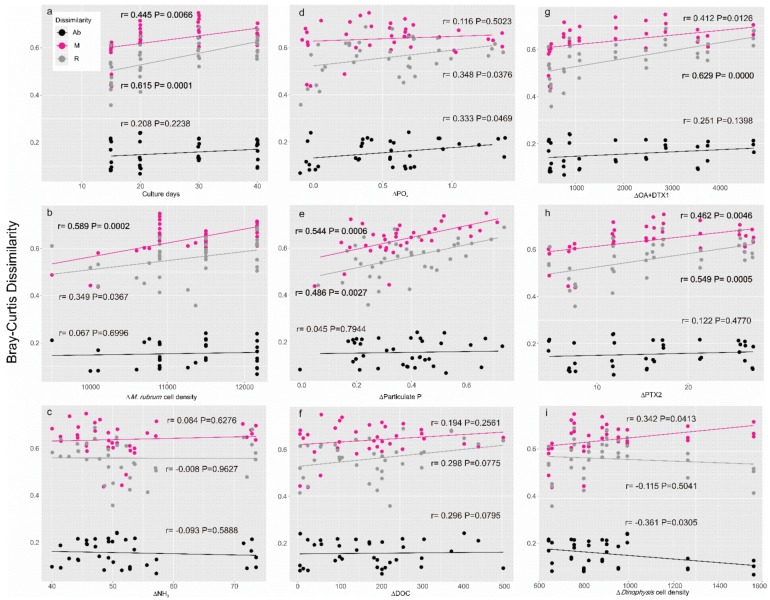
Bray–Curtis dissimilarities of the bacterial community between the onset (T1) and each of the following growth phases (T2–T5) against culture days (**a**) and variance of BIOENV-selected environmental factors (**b**–**i**) in the *Dinophysis* treatment.

**Figure 6:**
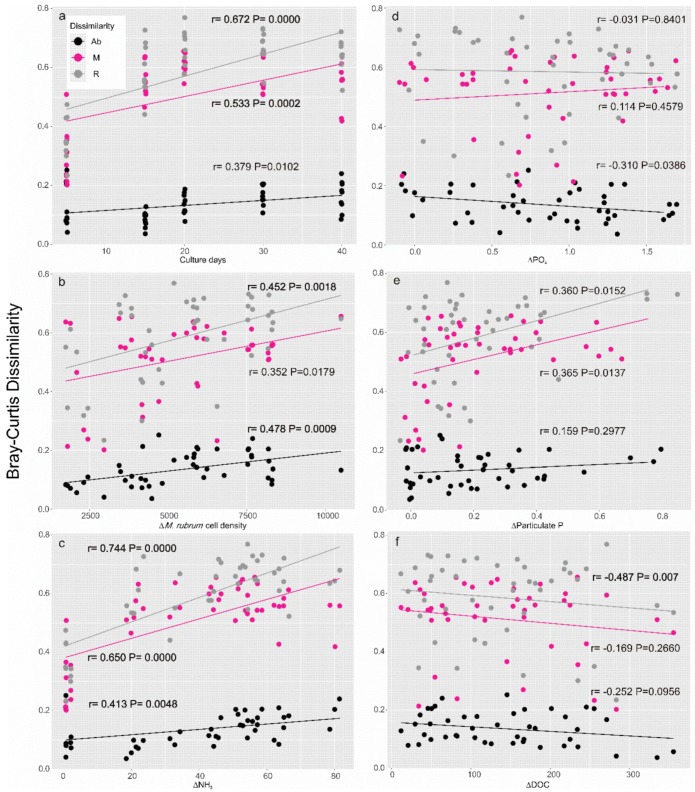
Bray–Curtis dissimilarities of the bacterial community between the onset (T0) and each of the following growth phases (T1–T5) against culture days (**a**) and variance of BIOENV-selected environmental factors (**b**–**f**) in the *M. rubrum* control.

Furthermore, Bray–Curtis dissimilarities were used to interpret the differences of the three assemblages of microbial communities during the growth phases of *Dinophysis* (Figure 5a) and *Mesodinium* (Figure 6a) and with the surrounding environment in a mixed culture (Figure 5b–i) as well as in a control treatment (Figure 6b–f). In detail, during *Dinophysis* growth (Group A), the dissimilarity between the M and R taxa varied significantly (*p* < 0.01) since the culture aged while the abundant taxa showed no differences (Figure 5a). As for the environmental factors, the M and R taxa also showed a more active response than the Ab taxa. These two assemblages positively correlated (*p* < 0.05) with changes of *M. rubrum* density (Figure 5b) and particulate organic phosphorus (Figure 5e) but showed no difference with ammonium (Figure 5c), dissolved the inorganic phosphate (PO_4_^3−^, Figure 5d), dissolved organic carbon (DOC, Figure 5f), or *Dinophysis* changes (Figure 5i). Interestingly, accumulation of both OA + DTX1 and PTX2 led to a significant partition of those bacterial taxa in moderate and low abundance (Figure 5g,h), which indicates the potential effects of the exposure to a high-toxin-concentration environment when the culture aged. The Ab taxa did not change with any of the environmental parameters. The response of the bacterial community in the *Mesodinium* control (Group B) was highly active. The correlations between Bray–Curtis dissimilarities of the bacterial community and days of cultivation were significant (*p* < 0.05) in all fractions (Figure 6a), *M. rubrum* cell density (Figure 6b), and variances of ammonium concentration (Figure 6c), which suggests that the bacterial community in the *M. rubrum* population, free of *Dinophysis*, would possibly be associated with the decline of the dominant species (*M. rubrum*) and biogeochemical characteristics mediated by the nutrient ammonium. Similar to the *Dinophysis*-*Mesodinium* co-culture treatment (Group A), PO_4_^3−^ (Figure 6e) showed a positive correlation with bacteria of the M and R taxa, which suggests the similar function of phosphate in the *Dinophysis* and *Mesodinium* predator-prey interaction. DOC may be critical in the variance of the bacterial community over time, but the interaction was limited to the R taxa (Figure 6f). The dominant prokaryotes in our artificial culture system were not sensitive to the changes of organic carbon levels.

**Figure 7:**
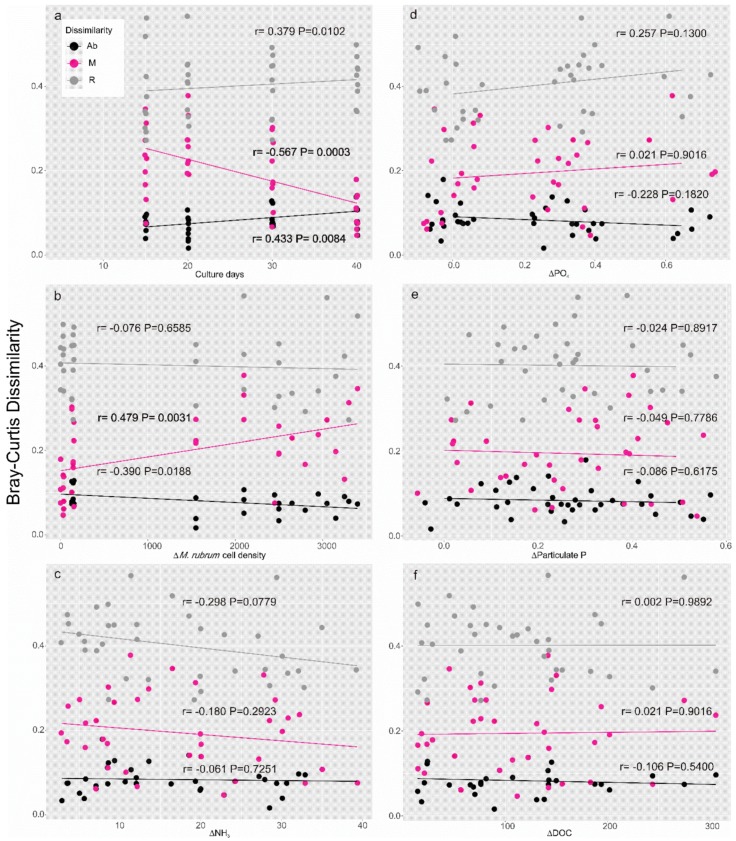
Bray–Curtis dissimilarities of the bacterial community between the two Groups at each growth phases (T2–T5) against the time of cultures (**a**) and variance of BIOENV-selected environmental factors (**b**–**f**).

Lastly, dissimilarities of the bacterial communities under the two treatments with the growth phases (Figure 7a) and all the selected factors (Figure 7b–f) were compared. Results of dissimilarities over time (Figure 7a) were consistent with the UniFrac distance plot (Figure 4c) where generalized distances generally implied OTUs of moderate abundance. Considering the resemblance of environmental factors between the two groups (Table 1), the significant decrease in distance of the 0.1–1% fraction (Figure 7a, r = −0.567, *p* = 0.0003) may be mostly attributed to the approaching environmental variables. Therefore, the previously detected deleterious effect of *Dinophysis* activity (Figure 5g,h) may be limited to the R taxa fraction in the culture medium. The differences of the selected factors did not appear to be responsible for the differences in the bacterial community (Figure 7c–f) except for the *M. rubrum* cell density effect with which the M taxa significantly varied (Figure 7b). The impact of *M. rubrum* cells on the M taxa was partly responsible for the decrease in dissimilarity over culture days (Figure 7a) and the largest differences of ciliate density were actually observed in the early phases of the *Mesodinium* growth curve (Figure 1). In summary, dissimilarities of the R taxa between the two groups (Figure 7) were generally higher than those of M and Ab taxas compared to the results within each group (Figure 5 and Figure 6), which leads to our assumption that the influence of *Dinophysis* and the toxins it produces may affect those R bacterial taxa.

## 3. Discussion

The algal–bacterial interaction has been shown to have a critical effect on bloom dynamics, but much of the research on this subject has been limited to photosynthetic algal species [34,35,36]. Due to their mixotrophic nature, *Dinophysis* species need to ingest *Mesodinium*, which is the sole determined prey [37,38], for population growth. Field populations of the ciliate usually aggregate in the subsurface water layers and perform diurnal vertical migration [39]. At the same time, *Dinophysis* species normally represent a small proportion of the phytoplankton community with a minor importance in relation to major biogeochemical cycles [25,40,41]. Thus, *Dinophysis* blooms and the time period from *Dinophysis* initiation to the *M. rubrum* decline is difficult to capture [10]. In the current study, we simulated an ideal predator-prey microcosms study to estimate the biogeochemical consequences of their co-occurrence and identify the ecological niche of bacterial communities developed during *Dinophysis* growth and *Mesodinium* decline.

We found that the *M. rubrum-Dinophysis* system intensively altered the biogeochemical status of the culture medium (Table 1). *Dinophysis* feeding activities accelerated the decline of the *M. rubrum* population (Figure 1). The differences in microbial communities between Groups A and B were mainly ascribed to the presence of *Dinophysis* cells (up to 1300 cells per mL^−1^) and the accumulation of toxins or other compounds produced by *Dinophysis*. In addition, considering that the influence of *Dinophysis* was restricted to the taxa in relatively low abundance, the dissimilarity may mainly consist of intracellular or phycosphere bacteria given that *Dinophysis* cells could function as particulate vectors or as hosts of certain bacterial species [42]. This assumption was also shown by the increased particulate organic carbon in this study (Table 1) as well as previous experiments [14]. Furthermore, in a study on bacterial assemblages associated with *Dinophysis*, it was also found that the addition of *Dinophysis* culture filtrate caused no significant changes in their relative abundance while the prokaryotic genera directly associated with *Dinophysis* were found in the >20-μm size fraction [31]. Species-specific grazing or deleterious effects of *Dinophysis* cells may also gradually shape the structure of the bacterial community. In a recent study [23], the potential contribution of bacterivory and bacterial remineralization to the growth of *Dinophysis* was calculated. It was concluded that neither of these processes were quantitatively relevant in order to support the increased biomass observed in the study.

Changes in nutrients and carbon content were observed in the *M. rubrum*–*D. acuminata* culture (Group A) (Figure 1 and Table 1), but the pattern was quite similar to of *M. rubrum* population dynamic itself (Group B, the control group), which indicates that ingestion by *Dinophysis* was unable to integrate most of the nutrients and carbon compounds derived from the *M. rubrum* population. Enhancement of secondary metabolite (DSP toxins) production was verified in *Dinophysis* cells [21] and in heterotrophic microbes [32] when exposed to *M. rubrum* living cells as well as cell lysate, but no direct evidence has emerged to decipher the associations among *Dinophysis*, microbes, and detritus in terms of nutrient cycling [37,43]. Tong et al. [14] estimated that *D. acuminata* was able to assimilate 65% and 25% of the particulate nitrogen and phosphate through predation, respectively. In the current study, the ingestion rate (ca. 3.25 prey cells predator^−1^ day^−1^) approached the highest level of the calculated growth rate where the predator: prey ratio was considered saturated [44]. The ingestion rate may not able to reach a higher value due to the low growth rate of the prey. However, the amount of ingested carbon calculated from a recently published paper [15] was about 2466.7 pg C cell^−1^ d^−1^, which is already beyond *Dinophysis* needs to maintain growth [8]. Field studies also found that the *Dinophysis* population preferred savaging on their prey in a short period of time [10,45].

After the *M. rubrum* population collapsed, the nutritional status of the culture medium was remarkably affected, which is expressed by the elevated levels of prey ammonium and fluctuant DOC and forms a hotspot of biogeochemical activities for the *Dinophysis* [40,46] and also the heterotrophic bacteria therein [47,48,49,50]. In this case, ammonium became the key component in the nitrogen cycle *Mesodinium* decline. NH_4_^+^ concentration remained constant until the addition of *Dinophysis* (T1, Table 1). A sharp increase was noticed due to a possible consequence of bacterial ammonification when *M. rubrum* decayed (Figure 1). Then NH_4_^+^ was significantly low at the late plateau phase of *Dinophysis* (T4 and T5, Table 1, Mann–Whitney Rank Sum Test, *p* = 0.041) when compared to the *Mesodinium* control. This finding is consistent with previous results showing that ammonium may lead to an increase of field populations of *Dinophysis* [51] and could be assimilated, which enhances the growth of *Dinophysis* at certain levels [23]. As for the other forms of nitrogen, nitrate with a moderate level (~50 μM) was dominant in our initial culture system but was used up in the first five days (Table 1, phase T1). When a high concentration of nitrate, e.g., up to 200 μM, is available in the culture medium, the availability of ammonium at lower concentrations (<2 μM) may be undetected or compensated [14]. The decay of the *M. rubrum* population seems unrelated to the nutrient limitation given the availability of both nitrogen (ammonium and nitrate) and phosphate in the culture medium [32,37].

Characterized as a plastidic-specific non-constitutive mixotroph [52], *Dinophysis* cells mainly retain chloroplasts from their prey and perform photosynthesis by those kleptoplastids as a carbon source. At the same time, marine heterotrophic bacteria play a major role in incorporating, respiring, and degrading dissolved organic carbon. However, changes of DOC concentration in this study hardly demonstrated the dissimilarities of the microbial community (Figure 5, Figure 6 and Figure 7), which suggests that the heterotrophic bacteria assemblages in our culture system may exhibit a metabolic versatility at least within the range of our DOC measurement. Moreover, bacterial communities at large phylogenetic group levels may exhibit general outcomes when exposed to high DOC concentrations [24]. Therefore, variation of DOC during phytoplankton dynamics may not taxonomically drive the shift of the major microbial community. Bacterial assemblages of M taxa (>0.1% and <1% of relative abundance) seem to be the most sensitive portion to changing the environmental nutrient conditions (Figure 5, Figure 6 and Figure 7). High-throughput sequencing revealed that this portion is composed of bacterial taxa assigned to the Rhodospirillaceae, Cytophagaceae, Flavobacteriaceae, and CHAB-XI-27 at family-level resolution. According to the 16s rDNA sequencing, Proteobacteria (Alphaproteobacteria-Rhodobacterales and Gammaproteobacteria-Cellvibrionales dominated) and Bacteroidetes (Sphingobacteria-Sphingobacteriales dominated) constituted more than 90% of the relative abundance of the microbial community in the culture medium cumulatively. These results are not surprising since only a limited number of heterotrophic bacterial lineages dominate those eukaryotic phytoplankton-associated communities [48]. Furthermore, these bacteria lineages cover those groups responsible for both monomer (such as amino acids) and polymer (such as chitin and protein) degradation in the ocean [53]. Less than 100 bacterial OTUs were assigned in our culture system, which is far less than previous field studies [45,54]. Bacterial community results from laboratory cultures show a lower diversity of bacterial assemblages because long-term maintenance may have eliminated those species that had already been overwhelmed and laboratory studies could avoid invasion of accidental species that are common in field studies. This is also the reason for which some studies exclude those rare species in their analysis [54]. The only study addressing the interaction between a *Dinophysis* bloom and the microbial community revealed that, even during the peak of a *Dinophysis acuminata* bloom (cell density ~1300 cell mL^−1^), the *Dinophysis* cells only accounted for 29% of the phytoplankton community [31]. Thus, the comparison of the bacterial community between our two groups could merely be attributed to the influence of the *D. acuminata* population. Our finding that only a low abundance of bacterial species was altered during *Dinophysis* intensive feeding activities indicated that, even though intense mixotrophy could remarkably drive biogeochemical dynamics, changes of the phytoplankton population may not be reflected by changes in those abundant bacterial phylotypes or in metabolic generalists. Moreover, connections may exist between specific species of bacteria and *Dinophysis* cells. Locating these connections by using metabolite and meta-transcriptome analysis may give us a further understanding for how these organisms interact with each other. Establishing an axenic culture of *Dinophysis* and comparing physiologies with non-axenic cultures over long-term periods may offer more robust evidence of the dependence of *Dinophysis* on bacteria [25]. However, attempts to purify *Dinophysis* and *M. ruburm* cells by using antibiotic treatment were not successful either in our laboratory or after efforts devoted by other groups [31]. Novel approaches to generate axenic algae cultures have been tested on the cyst-forming species *Gymnodinium catenatum* starting from resting cysts [55] and freshwater species by using fluorescence-activated cell sorting [56]. Yet, the methods proposed may not be universal and transferable considering that *Dinophysis* and *M. rubrum* cells are oddly shaped and fragile. More efforts are needed in the future to come up with appropriate approaches to initiate axenic *Dinophysis* cultures or elegant methods to directly target specific interactions between *Dinophysis* and associated bacteria.

## 4. Materials and Methods

### 4.1. Cultures

A unicellular algal culture of *D. acuminata* (DAYS01) was established from cells previously isolated from Xiaoping Island (121.53° E 38.83° N), the Yellow Sea, China in July 2014 [13]. The ciliate *M. rubrum* (AND-A0711) and the cryptophyte, *Teleaulax amphioxeia* (AND-A0710) were isolated from coastal waters off Huelva, Southern Spain in 2007 [57]. All cultures were routinely inoculated based on the cryptophyte–*M. rubrum*–*Dinophysis* food chain [7,13] in f/6-Si medium, which was prepared with 1/3 nitrate, 1/3 phosphate, 1/3 metals, and 1/3 of the vitamins concentrations in the f/2-Si medium. Cultures were maintained at 15 °C under a light intensity of 3000 lux and a 14 h light:10 h dark photo cycle.

### 4.2. Batch Culture Setup

A mono-algal culture of *M. rubrum* was maintained for a few days and gradually eaten by *Dinophysis* cultures. High ambient nutrient concentrations may obscure the detection of nutritional flows within the microbial loop. Therefore, *M. rubrum* were first inoculated from f/6 medium to f/10 and then to f/20. Then six *M. rubrum* replicates with an initial concentration of 6000 cells mL^−1^ were prepared in 5-L glass flasks to start this batch culture experiment. *Dinophysis* cells were pre-starved over 14 days, filtered onto 15-μm Nitex sieves, and gently rinsed with fresh artificial seawater to minimize carryover free living bacteria. The cells were then re-suspended in 90 mL of artificial seawater. After 5 days of inoculation of the *M. rubrum* cultures, 30 mL of the previously rinsed *Dinophysis* were added into three out of the six flasks (Group A). The other three *M. rubrum* cultures with the addition of 30 mL of artificial seawater were designated as Group B or control. The whole experiment was conducted at 15 °C under a light intensity of 3000 lux on a 14 h light:10 h dark photo cycle.

*Dinophysis* and/or the ciliate subsamples were taken every 2 or 3 days and fixed with 3% (*v/v*) formalin solution for microscopic enumeration in a Sedgewick-Rafter counting chamber at 100× magnification. For bacterial analysis, the formalin-preserved samples (1 mL) were stained with 2 μL of 4′,6-diami-dino-2-phenylindole (DAPI) solution (1 mg mL^−1^) and filtered onto a black polycarbonate filter (pore size: 0.22 μm, diameter: 25 mm, Millipore, Burlington, MA, USA). Then the filters were gently removed onto a glass slide and observed at 600× by using fluorescence microscopy (DMi8, Leica Microsystems, Buffalo Grove, IL, USA) under UV excitation.

### 4.3. Nutrient Sample Collection and Preparation

The growth curve was manually divided into six different growth phases. Six sampling spots were set up to collect nutrient and toxin samples, which are, hereafter, referred to as T0—at the very beginning of the incubation, T1—early phase following inoculation of *Dinophysis*, T2—the middle of the exponential growth of *Dinophysis*, T3—the end of the exponential growth of *Dinophysis*, T4—the depletion of *M. rubrum*, and T5—the end of *Dinophysis* growth.

For nutrients, 30-mL culture medium were filtered through pre-combusted GF/F filters (25 mm, Whatman, Maidstone, UK) for particulate organic carbon (POC) and particulate organic phosphate (POP) collection, respectively. The filters for POC were dried in a 60 °C oven for 24 h, stored at −20 °C, and analyzed on an elemental Analyzer (EA3000, EuroVector S.p.A, Milan, Italy). The particulate phosphate was converted to orthophosphate (PO_4_^3−^) by first hydrolyzing it by the addition of 5 mL of 5% potassium persulfate and 10 mL of Milli-Q water and then autoclaving it (121 °C) for 20 min. The filtrate was used for quantifying the dissolved inorganic nutrients (DIN and DIP) and dissolved organic carbon (DOC). Nitrate, ammonium, and phosphate were analyzed by SKALAR SAN^++^ Autoanalyser (SKALAR, Breda, The Netherlands). DOC concentration was determined by using a TOC Analyzer (Multi C/N 3100, Analytik Jena, Jena, Germany). All analyses were conducted following the protocols of the manufacturers.

### 4.4. Toxin Analysis

*Dinophysis* cells and culture medium were separated for toxin analysis. Between 10 and 30 mL of culture medium was harvested and analyzed for toxins since the inoculation day (T1) of *Dinophysis* cells. The medium was kept in 50-mL centrifuge tubes and stored at −20 °C before extraction. Solid-phase extraction (SPE) was employed [13,58] for the extraction of cells or medium samples. The SPE column (Oasis HLB 60 mg, Waters, Milford, MA, USA) was preconditioned with 6 mL of methanol and 6 mL of Milli-Q water. Once the cells or medium samples were loaded, the cartridge was washed with 3 mL of Milli-Q water and then blow-dried and eluted with 1 mL of methanol to collect the toxins into an HPLC vial. Eluates from the samples were then heated at 40 °C in a heating block (HP-S016SY), dried under a stream of N_2_, and re-suspended in 1 mL of 100% methanol for toxin analysis.

Toxin analysis was performed on an UltiMate 3000 LC (Thermo Scientific^TM^ Dionex^TM^, Waltham, MA, USA) and an AB 4000 mass spectrometer system (SCIEX, Framingham, MA, USA) with electrospray ionization. PTX2 was analyzed in positive mode, while OA and DTX1 were analyzed in negative mode. Chromatographic separation was performed by using a Waters XBridgeTM C18 column (3.0 × 150 mm, 3.5-μm particle size) (Milford, MA, USA) at 40 °C for a negative mode. The mobile phase consisted of phase A, 0.05 *v/v* % ammonia hydroxide in water, and phase B, 0.05 *v/v* % ammonia hydroxide in 90% acetonitrile with a flow rate of 0.4 mL min^−1^ and 10 µL injection. A linear gradient elution from 10% to 90% B was run for 9 min, held for 3 min at 90% B, decreased to 10% B for 2 min, and held at 10% B for 4 min to equilibrate at the initial conditions before the next run. In a positive mode, a Waters XBridgeTM C18 column (2.1 × 50 mm, 2.5-μm particle size) at 25 °C was used for chromatographic separation. A linear gradient from 10% to 80% acetonitrile containing a constant concentration of buffer (2 mM ammonium formate and 50 mM formic acid) was run between 0 min and 9 min and held at 80% acetonitrile for 2 min at a flow rate of 0.3 mL min^−1^. Standards for OA, DTX1, and PTX2 were purchased from the National Research Council, Canada.

### 4.5. DNA Extraction and Illumine Sequencing

At each time spot, a 120 to 150 mL culture medium was retrieved and filtered by using a 0.22-μm cellulose filter. Then the filters were folded and stored at −80 °C. Total genome DNA was extracted by using GenJET Genomic DNA Purification Kits (Thermo Scientific, Waltham, WA, USA) following the protocols of the manufacturer. Bacterial amplicons were produced by targeting the 16S V3-V4 hypervariable region with universal primers 343F (5′-TACGGRAGGCAGCAG-3′) and 798R (5′-AGGGTATCTAATCCT-3′). The amplicon quality was visualized by using gel electrophoresis, which was purified with AMPure XP beads (Agencourt) and amplified for another round of PCR. After being purified with the AMPure XP beads again, the final amplicon was quantified by using Qubit dsDNA assay kits. Equal amounts of purified amplicons were pooled for subsequent sequencing. The amplicon libraries were then sequenced on an Illumina MiSeq platform (Shanghai OE Biotechnology Co., Ltd., Shanghai, China).

### 4.6. Bioinformatics Analysis

Paired-end reads were preprocessed by using Trimmomatic software [59] to detect and cut off the ambiguous bases (N), barcodes, primers, and low-quality sequences. After trimming, paired-end reads were assembled by using FLASH [60]. Valid tags were subjected to clustering to generate operational taxonomic units (OTUs) using VSEARCH software (Version 2.4.2) at a 97% similarity setting [61]. The representative sequence of each OTU was selected by using the QIIME package. Representative reads were annotated and blasted against the Silva database (Version 123) using the ribosome database project (RDP) classifier with a confidence threshold of 70%. Sequences were submitted to the NCBI Sequence Read Archive with the accession number SRR6048156.

Package *phyloseq* [62] in R (http://www.Rproject.org, v. 3.3.3) was used to perform alpha and beta diversity calculations and to visualize the results of dimensional reduction approaches (nonmetric multidimensional scaling, NMDS). UniFrac distance matrices (weighted, unweighted, and generalized) were calculated with the R package *GUniFrac* [63] based on the OTU table and the phylogenetic tree. Note that the OTU table was square-root transformed and the explanatory matrix was z-score-transformed in R before the statistic procedures. To characterize the potential functions of different bacteria groups, we defined “abundant” (Ab) and “rare” (R) OTUs with the criteria of the average relative abundance across all the samples above 1% and below 0.1%, respectively [64]. The rest of the bacterial lineages (>0.1% and <1%) were assigned to a “moderately abundant” (M) group. The BIOENV procedure was implemented to identify the subset of a set of explanatory variables (nutrients profile, toxin content, and mixotrophs density). The Euclidean distance matrix of this correlates maximally with the Bray–Curtis compositional dissimilarity matrix of the OTU table. The Mantel test was also run with 999 permutations to check whether the subset of explanatory variables was able to capture the variation of bacterial communities from the three fractions. The BIOENV procedure and Mantel test on the three matrices (abundant, moderately-abundant, and rare fraction) were achieved by using relevant functions in the R package *vegan* [65]. Bray–Curtis dissimilarities of abundant (Ab), moderately abundant (M), and rare taxa (R) among samples were plotted against differences of explanatory variables (subtraction between samples) selected via BIOENV.

## Figures and Tables

**Table 1:** Summary of environmental variables in the *Dinophysis* + *Mesodinium* culture (A) and in the *Dinophysis*-free control (B) over the predator-prey growth curves. Data are presented as mean ± SD. POP and POC stand for particulate organic phosphate and particulate organic carbon, respectively.

Samples	POP (μM)	DIP (PO_4_^3−^) (μM)	DIN (NH4^+^) (μM)	DIN (NO_3_^−^) (μM)	DOC (μM)	POC (μM)	OA + DTX1 (pg mL^−1^)	PTX2 (ng mL^−1^)
T0	1.21 ± 0.05	2.90 ± 0.85	2.86 ± 2.02	45.95 ± 5.77	633.61 ± 74.23	792.80 ± 133.67	-	-
T1	1.26 ± 0.06	2.80 ± 0.37	2.62 ± 0.82	9.76 ± 5.77	977.85 ± 283.32	789.13 ± 124.75	99.33 ± 5.71	0.43 ± 0.03
T2 (A)	1.51 ± 0.12	2.47 ± 0.19	54.29 ± 1.89	2.50 ± 2.53	606.24 ± 211.48	666.22 ± 22.08	559.88 ± 23.09	6.87 ± 1.46
T2 (B)	1.31 ± 0.32	2.26 ± 0.32	30.36 ± 6.89	0.48 ± 0.41	463.64 ± 118.71	659.62 ± 30.02	-	-
T3 (A)	1.68 ± 0.03	2.37 ± 0.37	62.62 ± 11.79	LOD	693.88 ± 124.99	661.13 ± 23.72	1001.08 ± 163.95	13.45 ± 2.11
T3 (B)	1.45 ± 0.14	2.26 ± 0.32	50.24 ± 4.76	0.71 ± 0.71	764.90 ± 56.27	580.10 ± 16.55	-	-
T4 (A)	1.75 ± 0.17	2.15 ± 0.19	50.36 ± 2.51	LOD	614.06 ± 111.31	739.42 ± 53.55	2494.61 ± 526.41	18.71 ± 2.74
T4 (B)	1.65 ± 0.25	2.15 ± 0.19	57.86 ± 2.58	2.86 ± 2.02	636.20 ± 69.36	510.67 ± 56.38	-	-
T5 (A)	1.63 ± 0.19	2.04 ± 0.19	55.48 ± 17.04	LOD	391.74 ± 63.33	866.42 ± 70.63	4109.58 ± 621.79	26.74 ± 0.73
T5 (B)	1.35 ± 1.16	2.04 ± 0.19	72.38 ± 9.10	LOD	479.49 ± 36.14	409.98 ± 137.61	-	-

**Table 2:** The validity of tags and alpha diversity indexes in *Dinophysis* present (A) and *Dinophysis*-free control (B) at the six growth phases (T0–T5). Triplicate subsamples were harvested for each treatment and phase. One subsample failed to produce the 16S rRNA gene amplification (“T1-2”).

Sample ID	Valid Tags	Valid%	Goods Coverage	OTU Counts	Simpson	Shannon Wiener	Chao1
T0-1	35,860	91.37%	0.9995	57	1.31	0.51	64.8
T0-2	37,205	89.24%	0.9995	79	1.75	0.56	83.4
T0-3	36,596	91.55%	0.9994	57	1.69	0.59	81.0
T1-1	25,396	85.29%	0.9996	81	1.93	0.57	85.0
T1-2	-	-	-	-	-	-	-
T1-3	38,153	92.66%	0.9994	49	1.24	0.50	67.2
T2-1 (A)	38,611	92.30%	0.9996	52	1.84	0.61	56.0
T2-2 (A)	34,985	90.78%	0.9998	51	1.99	0.64	52.3
T2-3 (A)	33,591	87.98%	0.9992	55	1.29	0.47	82.1
T2-1 (B)	34,373	89.63%	0.9995	54	1.65	0.56	63.8
T2-2 (B)	36,081	91.48%	0.9995	48	1.60	0.54	59.0
T2-3 (B)	35,202	87.22%	0.9996	40	1.71	0.57	46.0
T3-1 (A)	38,304	92.79%	0.9995	59	1.55	0.53	65.6
T3-2 (A)	35,826	91.63%	0.9996	43	1.25	0.44	49.0
T3-3 (A)	36,145	92.71%	0.9997	47	1.52	0.51	51.7
T3-1 (B)	37,516	91.71%	0.9998	42	1.38	0.49	43.9
T3-2 (B)	35,153	90.96%	0.9995	44	1.16	0.42	63.5
T3-3 (B)	36,480	91.28%	0.9996	41	1.56	0.51	44.3
T4-1 (A)	35,231	91.41%	0.9998	42	1.56	0.51	43.7
T4-2 (A)	37,869	90.60%	0.9995	47	1.68	0.53	66.5
T4-3 (A)	36,610	92.61%	0.9995	53	1.62	0.52	66.0
T4-1 (B)	38,995	93.12%	0.9997	47	1.37	0.47	50.5
T4-2 (B)	35,561	87.96%	0.9996	45	1.85	0.60	49.5
T4-3 (B)	34,364	90.38%	0.9996	47	1.36	0.43	56.2
T5-1 (A)	33,756	88.31%	0.9996	46	1.86	0.62	55.2
T5-2 (A)	37,201	88.99%	0.9995	51	1.66	0.54	64.0
T5-3 (A)	36,041	89.82%	0.9995	57	1.70	0.56	65.7
T5-1 (B)	36,348	91.57%	0.9993	48	1.42	0.46	86.3
T5-2 (B)	36,231	90.09%	0.9994	48	1.78	0.59	63.2
T5-3 (B)	29,843	86.83%	0.9995	70	1.88	0.56	77.3

**Table 3:** The Mantel test on the relationship between the bacterial community and environmental factors.

Mantel Test	Pearson Correlation		Spearman Correlation	
	Statistic r	*p* Value	Statistic r	*p* Value
Ab taxa	0.361	0.002	0.363	0.001
M taxa	0.593	0.001	0.575	0.001
R taxa	0.520	0.001	0.542	0.001

**Table 4:** BIOENV procedure on the relationship between the bacterial community and environmental factors.

BIOENV	Pearson Correlation	Parameters in Best Model	Spearman Correlation	Parameters in Best Model
Ab taxa	0.340	DOC, OA + DTX1, PTX2, *M. rubrum*, *Dinophysis*	0.287	PO_4_^3−^, DOC, OA + DTX1, *M. rubrum*, *Dinophysis*
M taxa	0.756	PO_4_^3−^, NH_4_^+^, OA + DTX1, PTX2, *M. rubrum*, *Dinophysis*	0.729	PO_4_^3−^, NH_4_^+^, OA + DTX1, PTX2, *M. rubrum*, *Dinophysis*
R taxa	0.661	POP, PO_4_^3−^, NH_4_^+^, PTX2, *M. rubrum*	0.670	POP, PO_4_^3−^, NH_4_^+^, PTX2, *M. rubrum*
